# Effect of Dapagliflozin on Accelerometer-Based Measures of Physical Activity in Patients With Heart Failure: An Analysis of the DETERMINE Trials

**DOI:** 10.1161/CIRCHEARTFAILURE.124.012349

**Published:** 2024-08-30

**Authors:** Kieran F. Docherty, Ruben Buendia Lopez, Folke Folkvaljon, Rudolf A. de Boer, Martin R. Cowie, Ann Hammarstedt, Dalane W. Kitzman, Mikhail N. Kosiborod, Anna Maria Langkilde, Barry Reicher, Michele Senni, Sanjiv J. Shah, Subodh Verma, Scott D. Solomon, John J.V. McMurray

**Affiliations:** British Heart Foundation Cardiovascular Research Centre, University of Glasgow, Scotland, United Kingdom (K.F.D., J.J.V.M.).; Data Science, Late-Stage Development, Cardiovascular, Renal and Metabolic (R.B.L.), BioPharmaceuticals Research and Development, AstraZeneca, Gothenburg, Sweden.; Late Stage Development, Cardiovascular, Renal and Metabolism (A.H., A.M.L.), BioPharmaceuticals Research and Development, AstraZeneca, Gothenburg, Sweden.; Health Technology Assessment Statistics and Data Science, BioPharmaceuticals Business Unit, AstraZeneca, Barcelona, Spain (F.F.).; Department of Cardiology, Thoraxcenter, Erasmus Medical Center, Rotterdam, the Netherlands (R.A.d.B.).; Late-Stage Development, Cardiovascular, Renal and Metabolic, BioPharmaceuticals Research and Development, AstraZeneca, Boston, MA (M.R.C.).; Sections on Cardiovascular Medicine and Geriatrics/Gerontology, Wake Forest University School of Medicine, Winston-Salem, NC (D.W.K.).; Department of Cardiovascular Disease, Saint Luke’s Mid America Heart Institute, University of Missouri, Kansas City (M.N.K.).; AstraZeneca BioPharmaceuticals Research and Development, Late-Stage Development, Cardiovascular, Renal and Metabolic, Gaithersburg, MD (B.R.).; University of Milano-Bicocca, Cardiovascular Department, Papa Giovanni XXIII Hospital, Bergamo, Italy (M.S.).; Division of Cardiology, Department of Medicine, Northwestern University Feinberg School of Medicine, Chicago, IL (S.J.S.).; Division of Cardiac Surgery, St Michael’s Hospital, University of Toronto, ON, Canada (S.V.).; Division of Cardiovascular Medicine, Brigham and Women’s Hospital, Boston, MA (S.D.S.).

**Keywords:** accelerometry, exercise, sodium-glucose transporter 2 inhibitors, walk test, wearable electronic devices

## Abstract

**BACKGROUND:**

Wearable accelerometers can quantify the frequency and intensity of physical activity during everyday life and may provide complementary data to established functional outcome measures on the effect of heart failure therapies on functional limitations.

**METHODS:**

In a voluntary substudy of the DETERMINE trials (Dapagliflozin Effect on Exercise Capacity Using a 6-Minute Walk Test in Patients With Heart Failure), patients wore a waist-worn triaxial accelerometer for as long as possible (ideally for 24 h/d for 7 days) at 3 points during the trial, between the screening visit and randomization (baseline data), and during weeks 8 and 14 to 16. Accelerometer outcomes included the change from baseline to week 16 in the total number of steps, time spent in light-to-vigorous physical activity, time spent in moderate-to-vigorous physical activity, movement intensity during walking, number of vector magnitude units‚ and total activity counts.

**RESULTS:**

Adequate baseline and week 16 accelerometer data were available for 211 of 817 (26%) randomized patients (defined as ≥10 hours of wear time for ≥3 days). Dapagliflozin had a favorable effect on the mean change from baseline at 16 weeks in the number of steps (between-group difference, 778 [95% CI, 240–1315]), time spent in moderate-to-vigorous physical activity (0.16 [95% CI, 0.03–0.29] hours), and in the mean vector magnitude units (25 [95% CI, 0.1–49] counts per minute). There were no between-group differences in the other accelerometer outcomes of interest.

**CONCLUSIONS:**

In this exploratory analysis of the DETERMINE trials, dapagliflozin had a beneficial effect on selected accelerometer-based measures of physical activity in patients with heart failure across the entire left ventricular ejection fraction spectrum, yet did not improve 6-minute walk distance, as previously reported. These data suggest that accelerometer-based measurements of everyday activity may provide complementary information to 6-minute walk distance and identify beneficial effects of treatment not detected by 6-minute walk distance.

**REGISTRATION:**

URL: https://www.clinicaltrials.gov; Unique identifiers: NCT03877237 and NCT03877224.

**WHAT IS NEW?**

- In patients with heart failure, wearable accelerometers can assess the degree of physical and functional limitations during day-to-day living.
- Wearable accelerometers can be used to evaluate the effect of a treatment on these limitations.
- In the placebo-controlled DETERMINE trials (Dapagliflozin Effect on Exercise Capacity Using a 6-Minute Walk Test in Patients With Heart Failure), the SGLT2 (sodium-glucose transporter 2) inhibitor dapagliflozin had a favorable effect on several accelerometer-based measures of physical activity in patients with heart failure across the ejection fraction spectrum.

**WHAT ARE THE CLINICAL IMPLICATIONS?**

- The finding of a beneficial effect of dapagliflozin on accelerometer-based measures of physical activity, in the context of no effect on 6-minute walk distance, suggests that accelerometers may be able to provide additive information on a treatment’s effect on functional limitations in patients with heart failure.

Impairment of physical activity and functional limitations characterize the syndrome of heart failure (HF).^1,2^ As well as improving morbidity and mortality outcomes, interventions that improve the degree of physical and functional limitation in daily life are highly valued by patients with HF and are acceptable to regulators for approval. Traditional methods of assessing physical and functional limitations include patient-reported outcome measures (eg, Kansas City Cardiomyopathy Questionnaire [KCCQ]), 6-minute walk distance (6MWD), and cardiopulmonary exercise testing.^3^ However, several therapies have demonstrated a beneficial effect on KCCQ scores (and morbidity/mortality outcomes) but have had an inconsistent or no effect on 6MWD or cardiopulmonary exercise testing outcomes such as peak oxygen consumption.^4–8^ Wearable accelerometers may provide complementary data on functional limitations, quantifying the frequency and intensity of physical activity during everyday life.^3,9,10^

In this exploratory analysis of the DETERMINE trials (Dapagliflozin Effect on Exercise Capacity Using a 6-Minute Walk Test in Patients With Heart Failure), we examined the effect of the SGLT2 (sodium-glucose transporter 2) inhibitor dapagliflozin on a range of accelerometer-based measures of physical activity in people with HF across the whole spectrum of left ventricular ejection fraction (LVEF).^8^

## METHODS

The DETERMINE double-blind, placebo-controlled, multicenter trials assessed the efficacy of 16 weeks of treatment with dapagliflozin 10 mg once daily, compared with placebo, in addition to standard care on the total symptom score and physical limitation scale of the KCCQ and 6MWD in 313 patients with HF with reduced ejection fraction (DETERMINE-Reduced [https://www.clinicaltrials.gov, NCT03877237]) and in 504 patients with HF with preserved ejection fraction (DETERMINE-Preserved [https://www.clinicaltrials.gov, NCT03877224]). Full details about the trial protocol and primary results of the DETERMINE trials have been published.^8^

### Data Sharing

Data underlying the findings described in this article may be obtained in accordance with AstraZeneca’s data-sharing policy described at https://astrazenecagrouptrials.pharmacm.com/ST/Submission/Disclosure

### Code Availability

Code for the analyses in this article can be shared on request with the corresponding author if data have been released following the data-sharing policy described at https://astrazenecagrouptrials.pharmacm.com/ST/Submission/Disclosure.

### Trial Patients

The main inclusion criteria were as follows: age ≥18 years (DETERMINE-Reduced) or ≥40 years (DETERMINE-Preserved); LVEF ≤40% (DETERMINE-Reduced) or >40% and evidence of structural heart disease (DETERMINE-Preserved); elevated natriuretic peptide levels; a diagnosis of HF for >2 months; and New York Heart Association functional classes II to IV. In addition, there was a minimum 6MWD requirement for eligibility.^8^

Exclusion criteria included systolic blood pressure <95 mm Hg; estimated glomerular filtration rate <25 mL/min·1.73 m^2^; type 1 diabetes; other conditions likely to prevent patient participation in the trial or greatly limit life expectancy; and any condition precluding exercise testing. The Ethics Committee of each participating institution approved the protocols, and all patients gave written informed consent.

### Accelerometer Substudy

In a voluntary substudy at 67 sites in 10 countries, patients wore a waist-worn triaxial accelerometer with a sampling frequency of 100 Hz (Dynaport MoveMonitor; McRoberts B.V., the Hague, the Netherlands) for as long as possible (ideally for 24 h/d for 7 days) at 3 points during the trial, between the screening visit and randomization (baseline data), and during weeks 8 and 14 to 16 (hereafter referred to week 16) following randomization. Adequate wear time for inclusion in the accelerometer substudy was prespecified as ≥10 hours of wear time between 6:00 am and 10:00 pm, for ≥3 days during the 7-day period.^11^

### Accelerometer Outcomes

Accelerometer outcomes included in this analysis were the total number of steps (excluding those while walking on stairs), time spent in light-to-vigorous physical activity (LVPA), time spent in moderate-vigorous physical activity (MVPA), number of vector magnitude units (VMUs)‚ movement intensity during walking (measured in milligravities [mg]), and the total number of activity counts during worn periods. The time spent in LVPA and MVPA (in hours) is an estimate of the total wear time spent with an energy expenditure ≥1.5 METs (ie, equivalent to nonsedentary activity) and ≥3.0 METs (activity at or above the intensity of brisk walking and household chores), respectively, calculated using a validated algorithm.^12^ The number of VMUs per minute refers to the mean of the square root of squared values along the vertical axis signal, converted from raw acceleration to counts per minute, and is considered a proxy for the intensity of physical activity.^13^ Movement intensity during walking (measured in mg [1 mg=9.81×10^−3^ ms^−2^]) was calculated as the root mean square of the sum of the values along the x, y, and z signals (*x* axis) during periods classed as walking (defined as at least 3 consecutive steps during a standing period).

### Statistical Analyses

In this exploratory analysis, the study cohort was defined as those patients in DETERMINE-Reduced and DETERMINE-Preserved with paired accelerometer data at baseline and week 16. These patients were pooled to form the DETERMINE-Pooled cohort. The change in these accelerometer measures from baseline to weeks 8 and 16 was calculated in both treatment groups in all participants with available data. For the purpose of this analysis, the change from baseline to week 16 in each accelerometer measure was considered as the primary outcome, with the change from baseline at week 8 as the secondary outcome. The effect of dapagliflozin compared with placebo was examined using ANCOVA adjusted for baseline value of the outcome of interest, LVEF, trial, age, sex, body mass index, NT-proBNP (N-terminal pro-B-type natriuretic peptide), New York Heart Association functional class, and geographic region. No imputation for missing data was performed. The treatment effect estimates were calculated overall in the DETERMINE-Pooled cohort and separately in DETERMINE-Reduced and DETERMINE-Preserved. In the primary results of the DETERMINE trials, all change data were censored at the site-reported date of the start of the COVID-19 pandemic in their region.^8^ We did not apply this data censoring for the present study to maximize the number of patients with available data for these exploratory analyses. As the analyses presented were not prespecified and are considered exploratory, no formal testing for statistical significance was performed. All analyses were performed independently by 2 different analysts, 1 using SAS (Statistical Analysis Software, version 9.4; SAS Institute Inc, Cary, NC) and the other using Python programming language (version Python 3.9.5-foss-2021a).

## RESULTS

Of the 817 patients enrolled in the DETERMINE trials (313 in DETERMINE-Reduced and 504 in DETERMINE-Preserved), adequate accelerometer data at both baseline and week 16 were available for 211 (26%) participants (75 in DETERMINE-Reduced and 136 in DETERMINE-Preserved), of whom 107 and 104 were randomized to dapagliflozin and placebo, respectively. Of these 211, 179 (85%) had available data at week 8 (90 and 89 were randomized to dapagliflozin and placebo, respectively). The number of patients with accelerometer data at specified time points during the trials is provided in Table S1.

### Baseline Characteristics

The baseline characteristics of the 211 patients in the accelerometer substudy are presented in Table 1. Patients included in the accelerometer substudy were generally representative of the whole DETERMINE trial population. The mean age was 71 years, the mean LVEF was 43%, and 32% of patients were female. The proportion of patients with LVEF ≤40% and >40% was 36% and 64%, respectively. Most patients were in the New York Heart Association functional class II (84%), and the median NT-proBNP was 861 pg/mL (interquartile range, 514–1591). Median KCCQ-total symptom score was 78.1 (interquartile range, 64.1–87.5), and median 6MWD was 336 m (interquartile range, 275–386). The proportion of patients with type 2 diabetes or atrial fibrillation was 37% and 51%, respectively. The majority of patients (91%) were treated with a renin-angiotensin system inhibitor alone (75%) or in combination with a neprilysin inhibitor (16%) and a β-blocker (90%). Pretrial use of a mineralocorticoid receptor antagonist was recorded in 55% of patients.

**Table 1.**
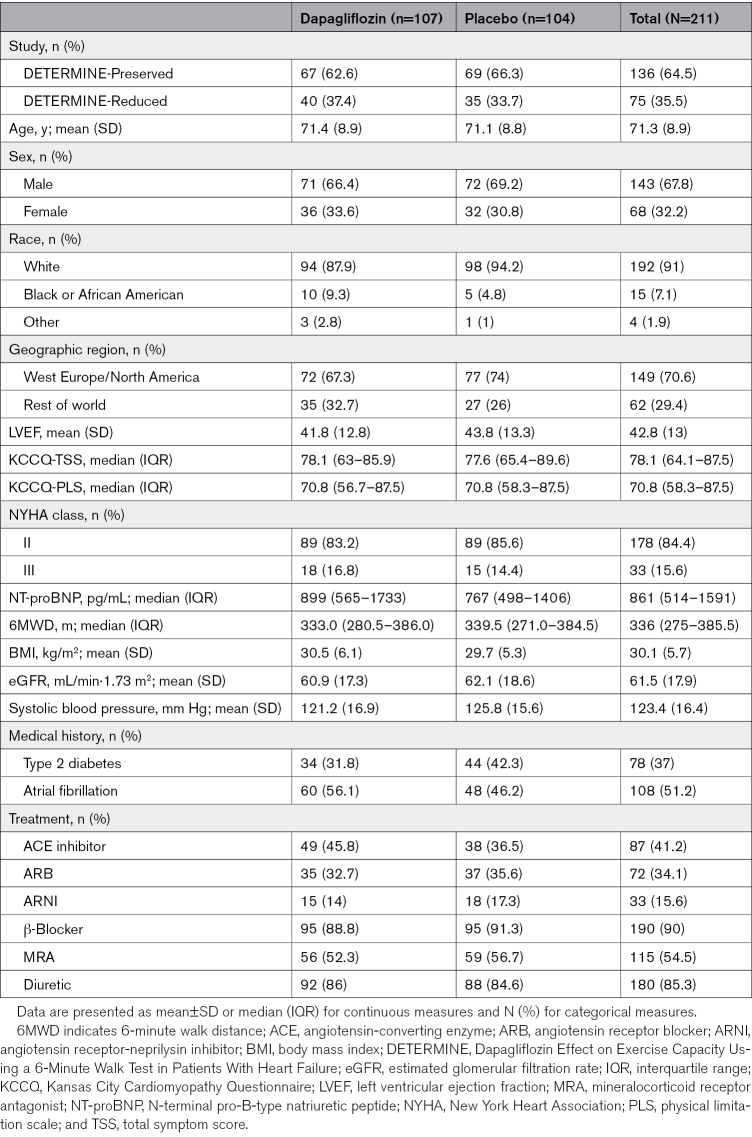
Baseline Characteristics in the DETERMINE-Pooled Accelerometer Substudy

### Effect of Dapagliflozin on Accelerometer Outcomes

The effect of dapagliflozin compared with placebo on the accelerometer-based outcomes of interest at weeks 8 and 16 in the DETERMINE-Pooled cohort is displayed in Tables 2 and 3, respectively, and the Figure. From baseline to week 8, levels of accelerometer-recorded physical activity declined in both treatment groups, with no significant between-group differences at week 8 following randomization.

**Table 2.**
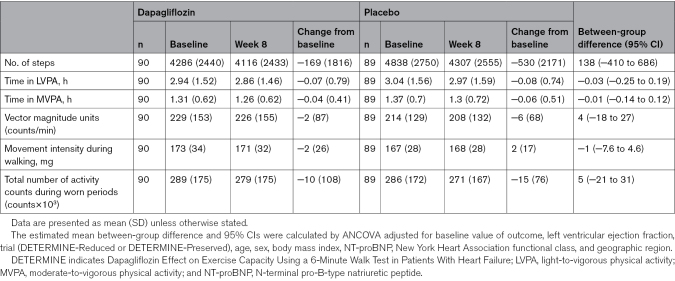
Effect of Dapagliflozin Compared With Placebo on Accelerometer Outcomes at Week 8

**Table 3.**
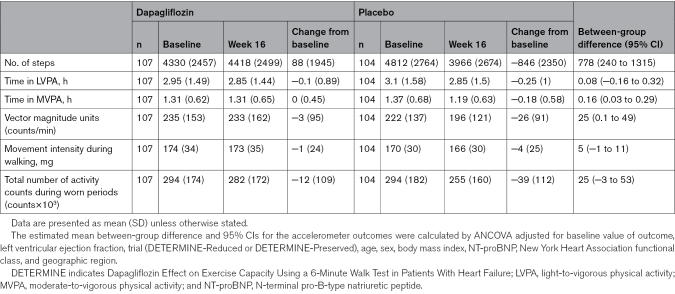
Effect of Dapagliflozin Compared With Placebo on Accelerometer Outcomes at Week 16

**Figure.**
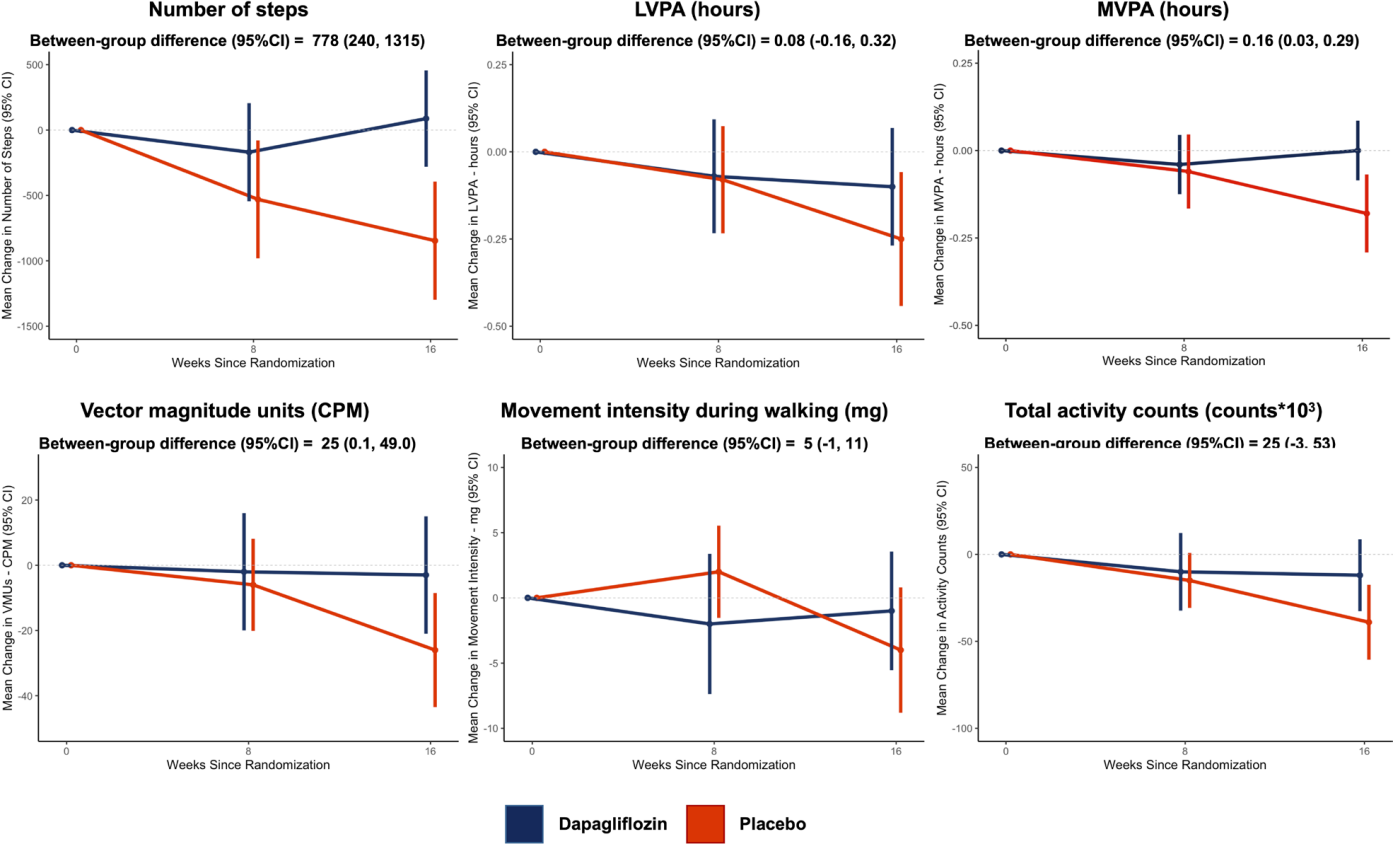
**Effect of dapagliflozin compared with placebo on the change from baseline at week 16 in accelerometer outcomes in DETERMINE-Pooled (Dapagliflozin Effect on Exercise Capacity Using a 6-Minute Walk Test in Patients With Heart Failure).** The estimated mean between-group differences and 95% CIs are the treatment effect estimates at week 16 and were calculated by ANCOVA adjusted for baseline value of outcome, left ventricular ejection fraction, trial (DETERMINE-Reduced or DETERMINE-Preserved), age, sex, body mass index, NT-proBNP (N-terminal pro-B-type natriuretic peptide), New York Heart Association functional class, and geographic region. CPM indicates counts per minute; LVPA, light-to-vigorous physical activity; mg, milligravities; MVPA, moderate-to-vigorous physical activity; and VMU, vector magnitude unit.

By week 16, measures of physical activity in the placebo group generally continued to fall, whereas, in the dapagliflozin group, there was an attenuation of the initial decline in the accelerometer-measured degree of physical activity. The mean change in the number of steps from baseline to week 16 was 88 (SD, 1945) and −846 (SD, 2350) in patients randomized to dapagliflozin and placebo, respectively; between-group difference was 778 (95% CI, 240–1315). The mean change in time spent in MVPA from baseline to week 16 was 0 (SD, 0.45) hours and −0.18 (SD, 0.58) hours in the dapagliflozin and placebo groups, respectively; between-group difference was 0.16 (95% CI, 0.03–0.29). The mean change in VMU (counts per minute) from baseline to week 16 was −3 (SD, 95) and −26 (SD, 91) in the dapagliflozin and placebo groups, respectively; between-group difference was 25 (95% CI, 0.1–49). There were no between-group differences in the time spent in LVPA, movement intensity during walking, or the total number of activity counts during worn periods.

The effect of dapagliflozin, compared with placebo, on accelerometer outcomes of interest in the 2 individual DETERMINE trials is displayed in Table S2 (DETERMINE-Reduced) and Table S3 (DETERMINE-Preserved). In DETERMINE-Reduced, the mean between-group difference in the number of steps was 920 (95% CI, 153–1688) in favor of dapagliflozin, with no other between-group differences in the other outcomes. In DETERMINE-Preserved, dapagliflozin had a favorable effect on time in LVPA (between-group difference, 0.31 [95% CI, 0.01–0.61] hours), MVPA (0.2 [95% CI, 0.03–0.37]), VMUs (between-group difference, 39 [95% CI, 4–75] counts per minute), and the total number of activity counts (between-group difference, 42 [95% CI, 3–81] counts×10^3^). In tests for a treatment effect interaction between the 2 trials, interaction *P* values for all outcomes were >0.10 except for time spent in LVPA (interaction *P*=0.01).

## DISCUSSION

In this exploratory pooled analysis of the DETERMINE-Reduced and DETERMINE-Preserved trials, 16 weeks of treatment with the SGLT2 inhibitor dapagliflozin had a beneficial effect on accelerometer-measured total daily number of steps, the quantity of MVPA, and VMUs (a measure of movement intensity). Taken together, these data suggest that 16 weeks of treatment with dapagliflozin had a favorable effect on both the quantity (number of steps and time in MVPA) and the intensity (VMUs) of physical activity during daily living among patients who took part in the voluntary accelerometer substudy of the DETERMINE trials and who provided adequate data for analysis.

To our knowledge, this is the first report of a pharmacological therapy for HF having a beneficial effect on accelerometer-based measures of physical activity. While a previous trial has reported on the negative effect of an intervention on an accelerometer-based measure of physical activity, no trials have reported the benefit of a therapy on an accelerometer-based outcome.^14^ The magnitude of the mean between-group difference in number of daily steps (778 [95% CI, 240–1315]) is similar to that suggested to be a minimally important clinical difference in patients with HF. For example, in an analysis of patients with HF in the National Health and Nutrition Examination Survey, each additional 1000 steps per day were associated with a 15% lower risk of all-cause mortality.^15^ Similar results have been reported in the general population with a 7% lower risk of cardiovascular mortality per 500 greater number of steps per day.^16^ The observed increase in time spent in MVPA of ≈10 minutes has previously been reported to be associated with a 5% lower risk of death or HF hospitalization in patients following implantation of implantable cardiac devices with activity-measuring capabilities.^17^ To our knowledge, no minimally important clinical difference in VMUs has been reported in patients with HF; however, the magnitude of the between-group difference of 25 cpm corresponds to an approximate 10% increase in the mean intensity of movement per minute.

SGLT2 inhibitors, as well as having consistent and meaningful benefits on reducing the risk of worsening HF and mortality, reduce patient-reported symptoms and improve the degree of patient-reported functional limitation as assessed by the KCCQ.^2,8,18–22^ Two other trials have examined the effect of SGLT2 inhibition on accelerometer outcomes. Neither the EMPIRE-HF trial (Empagliflozin in Heart Failure Patients With Reduced Ejection Fraction: A Randomized Clinical Trial) nor the CHIEF-HF trial (Canagliflozin: Impact on Health Status, Quality of Life and Functional Status in Heart Failure) reported a significant effect on accelerometer-measured daily activity counts or daily step counts, respectively.^23,24^ Direct between-trial comparisons are limited by several between-trial differences. First, the accelerometer devices used varied across trials, and this may have led to differences in the device’s sensitivity to detect low-level physical activity, as is common in patients with HF. Furthermore, differences in device placement (ie, whether waist- or wrist-worn) and the analytical methods used mean that the results for the same outcome (eg, steps) may not be directly comparable between trials. Second, the follow-up time in EMPIRE-HF and CHIEF-HF was shorter than that in the DETERMINE trials (12 versus 16 weeks). In DETERMINE, the significant between-group differences at 16 weeks were not present at 8 weeks, so a longer follow-up time may be required to detect a treatment’s effect on these outcomes. Finally, there may have been differences in the characteristics of the trial populations studied that influenced the results; there was a substantially lower level of baseline patient-reported health-related quality of life in CHIEF-HF as compared with the DETERMINE trials, and it is possible that this limited the ability of patients in CHIEF-HF to improve in terms of accelerometer-measured step count over the 12 weeks of follow-up.^24^

The benefits seen in this analysis of accelerometer outcomes contrast with the absence of a beneficial effect of dapagliflozin on 6MWD in each individual DETERMINE trial or in an exploratory pooled analysis of both trials (between-group difference, 2.5 [95% CI, −3.5 to 8.4] m), along with a lack of a consistent beneficial effect of SGLT2 inhibitors on 6MWD in several smaller trials, although in 1 of these (PRESERVED-HF [Dapagliflozin in PRESERVED Ejection Fraction Heart Failure]), dapagliflozin did increase 6MWD in a population that was more obese and had more severely impaired functional capacity at baseline, as discussed elsewhere.^6,8,25–30^ This discrepancy between an effect on accelerometer outcomes and 6MWD has been observed in other trials using different interventions. In the randomized WATCHFUL trial (Pedometer-Based Walking Intervention in Patients With Chronic Heart Failure With Reduced Ejection Fraction), a lifestyle walking intervention, combining self-monitoring with an activity tracker and telephone counseling, increased the step count by 1420 per day, the time spent in MVPA (+0.14 hours), and patient-reported health status, as compared with the control group, yet did not have any significant effect on the primary outcome of 6MWD (between-group difference, 7.4 [95% CI, −8.0 to 22.7] m).^31^ Notably, the effect of dapagliflozin on time spent in MVPA in DETERMINE-Pooled was the same as the effect of the lifestyle walking intervention in the WATCHFUL trial (0.16 versus 0.14 hours). In the NEAT-HFpEF trial (Nitrate’s Effect on Activity Tolerance in Heart Failure With Preserved Ejection Fraction), isosorbide mononitrate reduced accelerometer-measured physical activity but did not affect 6MWD, KCCQ scores, or NT-proBNP concentrations.^14,32^

Except for intravenous iron and cardiac resynchronization therapy, the majority of treatments for HF have had no consistent beneficial effect on 6MWD, despite favorable effects on patient-reported health-related quality of life and clinical outcomes.^33^ The data presented here from the DETERMINE trials and the results of the WATCHFUL and NEAT-HFpEF trials suggest that accelerometer-based measurements of everyday activity may provide complementary information to 6MWD and identify beneficial (or negative) effects of treatments or interventions not detected by 6MWD. Additional attractive features of the use of accelerometer-based measures as clinical trial outcomes include their ability to measure activity over longer periods during daily living, as opposed to a single time point as with commonly used assessments of functional capacity in trials such as 6MWD or peak oxygen consumption measured with cardiopulmonary exercise testing and that the measurements provided by accelerometers are more reflective of the intensity of activity at which patients with HF live for the majority of their time on a day-to-day basis.^9,10^

The lack of effect of dapagliflozin on 6MWD may reflect that the magnitude of dapagliflozin’s treatment effect on increasing step count, time spent in MVPA, and VMUs was insufficient to result in physiological adaptions, which translated into a measurable increase in an individual’s capacity for more metabolically demanding activity and an improved 6MWD. Although there are suggested mechanisms of action of SGLT2 inhibitors that could specifically improve aerobic exercise performance (eg, increase in erythropoiesis and hematocrit, enhanced skeletal muscle and myocardial iron utilization, and favorable cardiac remodeling), the magnitude of these effects and the limited training stimulus effect of an increase in steps and movement intensity may not have been sufficient to improve aerobic capacity (and 6MWD).^25,26,34,35^ Indeed, even in the HF-ACTION trial (Heart Failure: A Controlled Trial Investigating Outcomes of Exercise Training), structured aerobic exercise training at >60% of heart rate reserve resulted in only an ≈15-m increase in 6MWD at 6 months (ie, less than what is thought to be a clinically meaningful change) with no between-group difference at 12 months.^36^

Furthermore, noncardiac factors such as comorbidities that impair the oxygen cascade and are not modifiable by treatment with an SGLT2 inhibitor may have limited the scope for improvement in 6MWD, which for many patients with HF is not a test of submaximal exercise capacity (as is commonly thought) but often requires maximal or even supramaximal effort above anaerobic threshold.^37,38^ Nevertheless, the observed increase in step count, time spent in MVPA, and VMUs provide an objective validation for the improvements in patient-reported functional limitations previously reported with dapagliflozin.^2,19,20^

There are limitations to this exploratory post hoc analysis that should be recognized. The accelerometer substudy of the DETERMINE trials was voluntary; therefore, the patients who chose to take part and who provided adequate data for these analyses (26% of the total DETERMINE population) may not necessarily be representative of all patients with HF. Less than half of the patients with any accelerometry data provided adequate data for this study, highlighting a challenge with the use of wearable devices in clinical trials. Furthermore, the requirement for a minimum 6MWD for inclusion in the DETERMINE trials may limit the generalizability of the results. The definition of the amount of accelerometer data provided by a patient to be eligible for this study was the definition prespecified in the DETERMINE trials’ statistical analysis plan. We did not examine alternative data filter definitions, which may have provided alternative results. The use of a waist-worn device may have underestimated the time spent in stationary activities such as household chores, which may have been detected using a wrist-worn device. Wearable accelerometers may have limited sensitivity to detect low levels of activity as compared with more intense activity; however, the device used in the DETERMINE trials has been shown to perform favorably to other devices at lower levels of activity.^39^ Additionally, there are limitations to the use of categories of activity intensity that are not specific for patients with HF who expend more energy at any given level of activity as compared with patients without HF (and, therefore, the use of non–HF-specific cutoffs may underestimate the amount of activity).^40,41^ The small sample size may have limited our ability to detect smaller treatment effects on time in LVPA, movement intensity during walking, and the total number of activity counts. Due to the relatively short follow-up time and small number of clinical events, we were unable to examine the association between changes in accelerometer-based measures of physical activity and outcomes in the DETERMINE trials. Measurements relating to changes in cardiac function, filling pressures, and remodeling were not performed in the DETERMINE trials, so we were unable to examine the relationship between these and the observed changes in accelerometer-based measures of physical activity.

## CONCLUSIONS

In this exploratory analysis of the DETERMINE trials, dapagliflozin had a beneficial effect on selected accelerometer-based measures of physical activity in patients with HF across the entire LVEF spectrum. Dapagliflozin increased the total daily number of steps, the time spent in MVPA, and VMUs per minute (a measure of movement intensity) in patients with HF, yet it did not improve 6MWD, as previously reported. Accelerometer-based measurements of everyday activity may provide complementary information to 6MWD and identify beneficial effects of treatment not detected by 6MWD.

## ARTICLE INFORMATION

### Sources of Funding

The DETERMINE trials (Dapagliflozin Effect on Exercise Capacity Using a 6-Minute Walk Test in Patients With Heart Failure) were sponsored by AstraZeneca. Dr McMurray is supported by British Heart Foundation Centre of Research Excellence Grant RE/18/6/34217.

### Disclosures

Dr Docherty’s employer, the University of Glasgow, has been remunerated by AstraZeneca for his work on clinical trials; he has received speaker fees from AstraZeneca, Boehringer Ingelheim, Pharmacosmos, Translational Medical Academy, and Radcliffe Cardiology; has served on Advisory Boards for Us2.ai and holds stock in the company; has served on an advisory board and served on a clinical end point committee for Bayer AG; has performed consultancy for FIRE-1; and has received research grant support (paid to his institution) from AstraZeneca, Roche, Novartis, and Boehringer Ingelheim. Drs Buendia Lopez, Cowie, Hammarstedt, Langkilde, and Reicher and F. Folkvaljon are employees of AstraZeneca and may own stock or stock options. The institution of Dr de Boer has received research grants or fees from AstraZeneca, Abbott, Bristol Myers Squibb, Cardior Pharmaceuticals GmbH, NovoNordisk, and Roche; he has had speaker engagements with or received fees from or served on an advisory board for Abbott, AstraZeneca, Bristol Myers Squibb, Cardior Pharmaceuticals GmbH, NovoNordisk, and Roche; and has received travel support from Abbott, Cardior Pharmaceuticals GmbH, and Novo Nordisk. Dr Kitzman has been a consultant for AstraZeneca, Pfizer, Corvia Medical, Bayer, Boehringer Ingelheim, Novo Nordisk, Rivus, and St. Luke’s Medical Center; has received grant support from Novartis, AstraZeneca, Bayer, Pfizer, Novo Nordisk, Rivus, and St. Luke’s Medical Center; and owns stock in Gilead Sciences. Dr Kosiborod has served as a consultant or on an advisory board for 35Pharma, Alnylam, Amgen, Applied Therapeutics, AstraZeneca, Bayer, Boehringer Ingelheim, Cytokinetics, Dexcom, Eli Lilly, Esperion Therapeutics, Imbria, Janssen, Lexicon Pharmaceuticals, Merck (Diabetes and Cardiovascular), Novo Nordisk, Pharmacosmos, Pfizer, Sanofi, scPharmaceuticals, Structure Therapeutics, Vifor, and Youngene Therapeutics; has received research grants from AstraZeneca, Boehringer Ingelheim, and Pfizer; holds stocks in Artera Health and Saghmos Therapeutics; has received honoraria from AstraZeneca, Boehringer Ingelheim, and Novo Nordisk; and has received other research support from AstraZeneca. Dr Senni has received honoraria or consulting fees from Abbott, AstraZeneca, Bayer, Boehringer Ingelheim, Merck, MSD, Novartis, Novo Nordisk, and Vifor. Dr Shah was supported by research grants from the US National Institutes of Health (NIH; U54 HL160273; R01 HL140731; and R01 HL149423); has received research funding from AstraZeneca, Corvia, and Pfizer; and has received consulting fees from Abbott, Alleviant, Amgen, Aria CV, AstraZeneca, Axon Therapies, Bayer, Boehringer Ingelheim, Boston Scientific, BridgeBio, Bristol Myers Squibb, Corvia, Cytokinetics, Edwards Lifesciences, Eli Lilly, Eidos, Imara, Impulse Dynamics, Intellia, Ionis, Merck, NGM Biopharmaceuticals, Novartis, Novo Nordisk, Pfizer, Prothena, Regeneron, Rivus, Sardocor, Shifamed, Tenax, Tenaya, and Ultromics. Dr Verma holds a Tier 1 Canada Research Chair in Cardiovascular Surgery and reports receiving grants or research support or speaking honoraria from Amarin, Amgen, AstraZeneca, Bayer, Boehringer Ingelheim, Canadian Medical and Surgical Knowledge Translation Research Group, Eli Lilly, HLS Therapeutics, Humber River Health, Janssen, Merck, Novartis, Novo Nordisk, Pfizer, PhaseBio, S&L Solutions Event Management Inc, and Sanofi. Dr Solomon has received research grants from Actelion, Alnylam, Amgen, AstraZeneca, Bellerophon, Bayer, Bristol Myers Squibb, Celladon, Cytokinetics, Eidos, Gilead, GlaxoSmithKline, Ionis, Lilly, Mesoblast, MyoKardia, NIH/National Heart‚ Lung‚ and Blood Institute (NHLBI), Neurotronik, Novartis, NovoNordisk, Respicardia, Sanofi Pasteur, Theracos, and Us2.ai and has consulted for Abbott, Action, Akros, Alnylam, Amgen, Arena, AstraZeneca, Bayer, Boehringer Ingelheim, Bristol Myers Squibb, Cardior, Cardurion, Corvia, Cytokinetics, Daiichi Sankyo, GlaxoSmithKline, Lilly, Merck, Myokardia, Novartis, Roche, Theracos, Quantum Genomics, Cardurion, Janssen, Cardiac Dimensions, Tenaya, Sanofi Pasteur, Dinaqor, Tremeau, CellProThera, Moderna, American Regent, and Sarepta. Dr McMurray reports receiving support from British Heart Foundation Centre of Research Excellence Grant RE/18/6/34217 and the Vera Melrose Heart Failure Research Fund; payments through Glasgow University from work on clinical trials, consulting, and grants from Amgen, AstraZeneca, Bayer, Cardurion, Cytokinetics, GlaxoSmithKline and Novartis, British Heart Foundation, NIH/NHLBI, Boehringer Ingelheim, SQ Innovations, and Catalyze Group; personal consultancy fees from Alynylam Pharmaceuticals, Amgen, AnaCardio, AstraZeneca, Bayer, Berlin Cures, Bristol Myers Squibb, Cardurion, Cytokinetics, Ionis Pharmaceuticals, Novartis, Regeneron Pharmaceuticals, and River 2 Renal Corp; personal lecture fees from Abbott, Alkem Metabolics, Astra Zeneca, Blue Ocean Scientific Solutions Ltd, Boehringer Ingelheim, Canadian Medical and Surgical Knowledge, Emcure Pharmaceuticals Ltd, Eris Lifesciences, European Academy of CME, Hikma Pharmaceuticals, Imagica Health, Intas Pharmaceuticals, J.B. Chemicals and Pharmaceuticals Ltd, Lupin Pharmaceuticals, Medscape/Heart.Org, ProAdWise Communications, Radcliffe Cardiology, Sun Pharmaceuticals, The Corpus, Translation Research Group, and Translational Medicine Academy; and on the Data Safety Monitoring Board for WIRB-Copernicus Group Clinical Inc. He is a director of Global Clinical Trial Partners Ltd.

### Supplemental Material

Tables S1–S3

## Supplementary Material



## References

[1] O’Donnell J Smith-Byrne K Velardo C Conrad N Salimi-Khorshidi G Doherty A Dwyer T Tarassenko L Rahimi K. Self-reported and objectively measured physical activity in people with and without chronic heart failure: UK Biobank analysis. Open Heart. 2020;7:e001099. doi: 10.1136/openhrt-2019-001099 32153787 10.1136/openhrt-2019-001099 PMC7046950

[2] Butt JH Docherty KF Kosiborod MN Inzucchi SE Køber L Langkilde AM Martinez FA Bengtsson O Ponikowski P Sabatine MS. Dapagliflozin and physical and social activity limitations in heart failure with reduced ejection fraction. JACC Heart Fail. 2023;11:1411–1423. doi: 10.1016/j.jchf.2023.04.016 37318419 10.1016/j.jchf.2023.04.016

[3] Psotka MA Abraham WT Fiuzat M Filippatos G Lindenfeld J Ahmad T Felker GM Jacob R Kitzman DW Leifer ES. Functional and symptomatic clinical trial endpoints: the HFC-ARC scientific expert panel. JACC Heart Fail. 2022;10:889–901. doi: 10.1016/j.jchf.2022.09.012 36456063 10.1016/j.jchf.2022.09.012

[4] Piepoli MF Hussain RI Comin-Colet J Dosantos R Ferber P Jaarsma T Edelmann F. OUTSTEP-HF: randomised controlled trial comparing short-term effects of sacubitril/valsartan versus enalapril on daily physical activity in patients with chronic heart failure with reduced ejection fraction. Eur J Heart Fail. 2021;23:127–135. doi: 10.1002/ejhf.2076 33314487 10.1002/ejhf.2076

[5] Pieske B Wachter R Shah SJ Baldridge A Szeczoedy P Ibram G Shi V Zhao Z Cowie MR; PARALLAX Investigators and Committee members. Effect of sacubitril/valsartan vs standard medical therapies on plasma NT-proBNP concentration and submaximal exercise capacity in patients with heart failure and preserved ejection fraction: the PARALLAX randomized clinical trial. JAMA. 2021;326:1919–1929. doi: 10.1001/jama.2021.18463 34783839 10.1001/jama.2021.18463 PMC8596197

[6] Abraham WT Lindenfeld J Ponikowski P Agostoni P Butler J Desai AS Filippatos G Gniot J Fu M Gullestad L. Effect of empagliflozin on exercise ability and symptoms in heart failure patients with reduced and preserved ejection fraction, with and without type 2 diabetes. Eur Heart J. 2021;42:700–710. doi: 10.1093/eurheartj/ehaa943 33351892 10.1093/eurheartj/ehaa943

[7] Lewis GD Voors AA Cohen-Solal A Metra M Whellan DJ Ezekowitz JA Böhm M Teerlink JR Docherty KF Lopes RD. Effect of omecamtiv mecarbil on exercise capacity in chronic heart failure with reduced ejection fraction: the METEORIC-HF randomized clinical trial. JAMA. 2022;328:259–269. doi: 10.1001/jama.2022.11016 35852527 10.1001/jama.2022.11016 PMC9297119

[8] McMurray JJV Docherty KF de Boer RA Hammarstedt A Kitzman DW Kosiborod MN Maria Langkilde A Reicher B Senni M Shah SJ. Effect of dapagliflozin versus placebo on symptoms and 6-minute walk distance in patients with heart failure: the DETERMINE randomized clinical trials. Circulation. 2024;149:825–838. doi: 10.1161/CIRCULATIONAHA.123.065061 38059368 10.1161/CIRCULATIONAHA.123.065061

[9] Vetrovsky T Clark CCT Bisi MC Siranec M Linhart A Tufano JJ Duncan MJ Belohlavek J. Advances in accelerometry for cardiovascular patients: a systematic review with practical recommendations. ESC Heart Fail. 2020;7:2021–2031. doi: 10.1002/ehf2.12781 32618431 10.1002/ehf2.12781 PMC7524133

[10] Buendia R Karpefors M Folkvaljon F Hunter R Sillen H Luu L Docherty K Cowie MR. Wearable sensors to monitor physical activity in heart failure clinical trials: state-of-the-art review. J Card Fail. 2024;30:703–716. doi: 10.1016/j.cardfail.2024.01.016 38452999 10.1016/j.cardfail.2024.01.016

[11] Colley R Connor Gorber S Tremblay MS. Quality control and data reduction procedures for accelerometry-derived measures of physical activity. Health Rep. 2010;21:63–69. https://pubmed.ncbi.nlm.nih.gov/20426228/20426228

[12] van Hees VT van Lummel RC Westerterp KR. Estimating activity-related energy expenditure under sedentary conditions using a tri-axial seismic accelerometer. Obesity (Silver Spring). 2009;17:1287–1292. doi: 10.1038/oby.2009.55 19282829 10.1038/oby.2009.55

[13] CHMP/EMA/SAWP. Committee for Medicinal Products for Human Use, European Medicines Agency, Scientific Advice Working Party. Qualification of opinion on proactive in COPD. 2018.

[14] Redfield MM Anstrom KJ Levine JA Koepp GA Borlaug BA Chen HH LeWinter MM Joseph SM Shah SJ Semigran MJ; NHLBI Heart Failure Clinical Research Network. Isosorbide mononitrate in heart failure with preserved ejection fraction. N Engl J Med. 2015;373:2314–2324. doi: 10.1056/NEJMoa1510774 26549714 10.1056/NEJMoa1510774 PMC4712067

[15] Zhou Y Sun X Yang G Ding N Pan X Zhong A Guo T Peng Z Chai X. Sex-specific differences in the association between steps per day and all-cause mortality among a cohort of adult patients from the United States with congestive heart failure. Heart Lung. 2023;62:175–179. doi: 10.1016/j.hrtlng.2023.07.009 37541137 10.1016/j.hrtlng.2023.07.009

[16] Banach M Lewek J Surma S Penson PE Sahebkar A Martin SS Bajraktari G Henein MY Reiner Z Bielecka-Dąbrowa A. The association between daily step count and all-cause and cardiovascular mortality: a meta-analysis. Eur J Prev Cardiol. 2023;30:1975–1985. doi: 10.1093/eurjpc/zwad229 37555441 10.1093/eurjpc/zwad229

[17] Conraads VM Spruit MA Braunschweig F Cowie MR Tavazzi L Borggrefe M Hill MRS Jacobs S Gerritse B van Veldhuisen DJ. Physical activity measured with implanted devices predicts patient outcome in chronic heart failure. Circ Heart Fail. 2014;7:279–287. doi: 10.1161/CIRCHEARTFAILURE.113.000883 24519908 10.1161/CIRCHEARTFAILURE.113.000883

[18] Vaduganathan M Docherty KF Claggett BL Jhund PS de Boer RA Hernandez AF Inzucchi SE Kosiborod MN Lam CSP Martinez F. SGLT-2 inhibitors in patients with heart failure: a comprehensive meta-analysis of five randomised controlled trials. Lancet. 2022;400:757–767. doi: 10.1016/S0140-6736(22)01429-5 36041474 10.1016/S0140-6736(22)01429-5

[19] Kosiborod MN Jhund PS Docherty KF Diez M Petrie MC Verma S Nicolau JC Merkely B Kitakaze M DeMets DL. Effects of dapagliflozin on symptoms, function, and quality of life in patients with heart failure and reduced ejection fraction: results from the DAPA-HF trial. Circulation. 2020;141:90–99. doi: 10.1161/CIRCULATIONAHA.119.044138 31736335 10.1161/CIRCULATIONAHA.119.044138 PMC6964869

[20] Kosiborod MN Bhatt AS Claggett BL Vaduganathan M Kulac IJ Lam CSP Hernandez AF Martinez FA Inzucchi SE Shah SJ. Effect of dapagliflozin on health status in patients with preserved or mildly reduced ejection fraction. J Am Coll Cardiol. 2023;81:460–473. doi: 10.1016/j.jacc.2022.11.006 36526515 10.1016/j.jacc.2022.11.006

[21] Butler J Anker SD Filippatos G Khan MS Ferreira JP Pocock SJ Giannetti N Januzzi JL Piña IL Lam CSP; EMPEROR-Reduced Trial Committees and Investigators. Empagliflozin and health-related quality of life outcomes in patients with heart failure with reduced ejection fraction: the EMPEROR-reduced trial. Eur Heart J. 2021;42:1203–1212. doi: 10.1093/eurheartj/ehaa1007 33420498 10.1093/eurheartj/ehaa1007 PMC8014525

[22] Butler J Filippatos G Jamal Siddiqi T Brueckmann M Böhm M Chopra VK Pedro Ferreira J Januzzi JL Kaul S Piña IL. Empagliflozin, health status, and quality of life in patients with heart failure and preserved ejection fraction: the EMPEROR-preserved trial. Circulation. 2022;145:184–193. doi: 10.1161/CIRCULATIONAHA.121.057812 34779658 10.1161/CIRCULATIONAHA.121.057812 PMC8763045

[23] Jensen J Omar M Kistorp C Poulsen MK Tuxen C Gustafsson I Køber L Gustafsson F Faber J Fosbøl EL. Twelve weeks of treatment with empagliflozin in patients with heart failure and reduced ejection fraction: a double-blinded, randomized, and placebo-controlled trial. Am Heart J. 2020;228:47–56. doi: 10.1016/j.ahj.2020.07.011 32798787 10.1016/j.ahj.2020.07.011

[24] Spertus JA Birmingham MC Nassif M Damaraju CV Abbate A Butler J Lanfear DE Lingvay I Kosiborod MN Januzzi JL. The SGLT2 inhibitor canagliflozin in heart failure: the CHIEF-HF remote, patient-centered randomized trial. Nat Med. 2022;28:809–813. doi: 10.1038/s41591-022-01703-8 35228753 10.1038/s41591-022-01703-8 PMC9018422

[25] Santos-Gallego CG Vargas-Delgado AP Requena-Ibanez JA Garcia-Ropero A Mancini D Pinney S Macaluso F Sartori S Roque M Sabatel-Perez F; EMPA-TROPISM (ATRU-4) Investigators. Randomized trial of empagliflozin in nondiabetic patients with heart failure and reduced ejection fraction. J Am Coll Cardiol. 2021;77:243–255. doi: 10.1016/j.jacc.2020.11.008 33197559 10.1016/j.jacc.2020.11.008

[26] Lee MMY Brooksbank KJM Wetherall K Mangion K Roditi G Campbell RT Berry C Chong V Coyle L Docherty KF. Effect of empagliflozin on left ventricular volumes in patients with type 2 diabetes, or prediabetes, and heart failure with reduced ejection fraction (SUGAR-DM-HF). Circulation. 2021;143:516–525. doi: 10.1161/CIRCULATIONAHA.120.052186 33186500 10.1161/CIRCULATIONAHA.120.052186 PMC7864599

[27] Nassif ME Windsor SL Tang F Khariton Y Husain M Inzucchi SE McGuire DK Pitt B Scirica BM Austin B. Dapagliflozin effects on biomarkers, symptoms, and functional status in patients with heart failure with reduced ejection fraction: the DEFINE-HF trial. Circulation. 2019;140:1463–1476. doi: 10.1161/CIRCULATIONAHA.119.042929 31524498 10.1161/CIRCULATIONAHA.119.042929

[28] Nassif ME Windsor SL Borlaug BA Kitzman DW Shah SJ Tang F Khariton Y Malik AO Khumri T Umpierrez G. The SGLT2 inhibitor dapagliflozin in heart failure with preserved ejection fraction: a multicenter randomized trial. Nat Med. 2021;27:1954–1960. doi: 10.1038/s41591-021-01536-x 34711976 10.1038/s41591-021-01536-x PMC8604725

[29] Palau P Amiguet M Domínguez E Sastre C Mollar A Seller J Garcia Pinilla JM Larumbe A Valle A Gómez Doblas JJ; DAPA-VO2 Investigators (see Appendix). Short-term effects of dapagliflozin on maximal functional capacity in heart failure with reduced ejection fraction (DAPA-VO2): a randomized clinical trial. Eur J Heart Fail. 2022;24:1816–1826. doi: 10.1002/ejhf.2560 35604416 10.1002/ejhf.2560

[30] Lewis GD Gosch K Cohen LP Nassif ME Windsor SL Borlaug BA Kitzman DW Shah SJ Khumri T Umpierrez G. Effect of dapagliflozin on 6-minute walk distance in heart failure with preserved ejection fraction: PRESERVED-HF. Circ Heart Fail. 2023;16:e010633. doi: 10.1161/CIRCHEARTFAILURE.123.010633 37869881 10.1161/CIRCHEARTFAILURE.123.010633 PMC10655911

[31] Vetrovsky T Siranec M Frybova T Gant I Svobodova I Linhart A Parenica J Miklikova M Sujakova L Pospisil D; WATCHFUL Investigators. Lifestyle walking intervention for patients with heart failure with reduced ejection fraction: the WATCHFUL trial. Circulation. 2024;149:177–188. doi: 10.1161/CIRCULATIONAHA.123.067395 37955615 10.1161/CIRCULATIONAHA.123.067395 PMC10782943

[32] Snipelisky D Kelly J Levine JA Koepp GA Anstrom KJ McNulty SE Zakeri R Felker GM Hernandez AF Braunwald E. Accelerometer measured daily activity in heart failure with preserved ejection fraction: clinical correlates and association with standard heart failure severity indices. Circ Heart Fail. 2017;10:e003878. doi: 10.1161/CIRCHEARTFAILURE.117.003878 28588021 10.1161/CIRCHEARTFAILURE.117.003878 PMC5634329

[33] Olsson LG Swedberg K Clark AL Witte KK Cleland JGF. Six minute corridor walk test as an outcome measure for the assessment of treatment in randomized, blinded intervention trials of chronic heart failure: a systematic review. Eur Heart J. 2005;26:778–793. doi: 10.1093/eurheartj/ehi162 15774495 10.1093/eurheartj/ehi162

[34] Docherty KF Curtain JP Anand IS Bengtsson O Inzucchi SE Køber L Kosiborod MN Langkilde AM Martinez FA Ponikowski P; DAPA-HF Investigators and Committees. Effect of dapagliflozin on anaemia in DAPA-HF. Eur J Heart Fail. 2021;23:617–628. doi: 10.1002/ejhf.2132 33615642 10.1002/ejhf.2132 PMC11497230

[35] Docherty KF Welsh P Verma S De Boer RA O’Meara E Bengtsson O Køber L Kosiborod MN Hammarstedt A Langkilde AM; DAPA-HF Investigators and Committees. Iron deficiency in heart failure and effect of dapagliflozin: findings from DAPA-HF. Circulation. 2022;146:980–994. doi: 10.1161/CIRCULATIONAHA.122.060511 35971840 10.1161/CIRCULATIONAHA.122.060511 PMC9508991

[36] O’Connor CM Whellan DJ Lee KL Keteyian SJ Cooper LS Ellis SJ Leifer ES Kraus WE Kitzman DW Blumenthal JA; HF-ACTION Investigators. Efficacy and safety of exercise training in patients with chronic heart failure: HF-ACTION randomized controlled trial. JAMA. 2009;301:1439–1450. doi: 10.1001/jama.2009.454 19351941 10.1001/jama.2009.454 PMC2916661

[37] Faggiano P D’Aloia A Gualeni A Lavatelli A Giordano A. Assessment of oxygen uptake during the 6-minute walking test in patients with heart failure: preliminary experience with a portable device. Am Heart J. 1997;134:203–206. doi: 10.1016/s0002-8703(97)70125-x 9313598 10.1016/s0002-8703(97)70125-x

[38] Mapelli M Salvioni E Paneroni M Gugliandolo P Bonomi A Scalvini S Raimondo R Sciomer S Mattavelli I La Rovere MT. Brisk walking can be a maximal effort in heart failure patients: a comparison of cardiopulmonary exercise and 6 min walking test cardiorespiratory data. ESC Heart Fail. 2022;9:812–821. doi: 10.1002/ehf2.13781 34970846 10.1002/ehf2.13781 PMC8934957

[39] Storm FA Heller BW Mazzà C. Step detection and activity recognition accuracy of seven physical activity monitors. PLoS One. 2015;10:e0118723. doi: 10.1371/journal.pone.0118723 25789630 10.1371/journal.pone.0118723 PMC4366111

[40] Schwendinger F Wagner J Infanger D Schmidt-Trucksäss A Knaier R. Methodological aspects for accelerometer-based assessment of physical activity in heart failure and health. BMC Med Res Methodol. 2021;21:251. doi: 10.1186/s12874-021-01350-6 34775952 10.1186/s12874-021-01350-6 PMC8590791

[41] Blasco-Peris C Climent-Paya V Vetrovsky T García-Álvarez MI Manresa-Rocamora A Beltrán-Carrillo VJ Sarabia JM. International Physical Activity Questionnaire Short Form and accelerometer-assessed physical activity: concurrent validity using six cut-points in HF patients. ESC Heart Fail. 2024;11:126–135. doi: 10.1002/ehf2.14514 37842962 10.1002/ehf2.14514 PMC10804186

