# Supplementary material for: Effect of Dapagliflozin on Accelerometer-Based Measures of Physical Activity in Patients With Heart Failure: An Analysis of the DETERMINE Trials
Source: Circ Heart Fail. 2024 Aug 30;17(10):e012349. doi: 10.1161/CIRCHEARTFAILURE.124.012349 (PMC11472896; doi:10.1161/CIRCHEARTFAILURE.124.012349)
Supplement: Supplementary file 1 [file hhf-17-e012349-s001.pdf]

The effect of dapagliflozin on accelerometer-based measures of physical activity in patients with heart failure: an analysis of the DETERMINE trials

Supplementary material

Supplementary Table 1: Number of patients with available accelerometer data

	Baseline	Week 8	End-of-study
Patients randomized and still in trial at timepoint, n (%)	817 (100)	807 (99)	797 (98)
Patients with any accelerometer data at specified timepoints, n (%)	448 (55)	450 (55)	385 (47)
Patients with sufficient* accelerometer data at specified timepoints, n (%)	319 (39)	364 (45)	319 (39)
Patients with sufficient* accelerometer data at specified timepoints and all earlier timepoints, n (%)	319 (39)	231 (28)	179 (22)

* Sufficient defined as ≥10 hours for ≥3 days

The denominator is the total number of randomized patients (N=817).

Supplementary Table 2: Effect of dapagliflozin compared with placebo on accelerometer outcomes at Week 16 in DETERMINE-Reduced

	Dapagliflozin				Placebo				
	**n=**	**Baseline**	**Week 16**	**Change from baseline**	**n=**	**Baseline**	**Week 16**	**Change from baseline**	**Between-group difference (95% CI)**
**Number of steps**	**40**	3922 (1955)	4169 (2053)	247 (1400)	**35**	5079 (2735)	3900 (2919)	-1179 (2258)	920 (153, 1688)
**Time in LVPA (hours)**	**40**	2.7 (1.48)	2.45 (1.16)	-0.25 (0.88)	**35**	3.18 (1.67)	3.07 (1.81)	-0.1 (1.07)	-0.38 (-0.78, 0.02)
**Time in MVPA (hours)**	**40**	1.22 (0.6)	1.2 (0.58)	-0.02 (0.36)	**35**	1.38 (0.67)	1.23 (0.73)	-0.16 (0.55)	0.05 (-0.13, 0.26)
**Vector magnitude units (counts per minute)**	**40**	184 (104)	177 (94)	-6 (60)	**35**	227 (119)	204 (128)	-23 (75)	-1 (-31, 30)
**Movement intensity during walking (milligravities)**	**40**	174 (32)	171 (36)	-3 (22)	**35**	174 (30)	169 (32)	-4 (32)	2 (-9, 13)
**Total number of activity counts during worn periods (counts*10^3)**	**40**	239 (136)	224 (118)	-15 (82)	**35**	307 (163)	274 (174)	-32 (96)	-7 (-45, 31)

Data presented as mean (SD) unless otherwise stated.

The estimated mean between-group difference and 95% confidence intervals were calculated by ANCOVA adjusted for baseline value of outcome, left ventricular ejection fraction, age, sex, body mass index, NT-proBNP, New York Heart Association functional class and geographic region.

Abbreviations: LVPA, light-to-vigorous physical activity; MVPA, moderate-to-vigorous physical activity.

Supplementary Table 3: Effect of dapagliflozin compared with placebo on accelerometer outcomes at Week 16 in DETERMINE-Preserved

	Dapagliflozin				Placebo				
	**n=**	**Baseline**	**Week 16**	**Change from baseline**	**n=**	**Baseline**	**Week 16**	**Change from baseline**	**Between-group difference (95% CI)**
**Number of steps**	**67**	4574 (2697)	4567 (2735)	-7 (2213)	**69**	4676 (2788)	4000 (2562)	-676 (2393)	632 (-90, 1355)
**Time in LVPA (hours)**	**67**	3.11 (1.48)	3.09 (1.53)	-0.02 (0.89)	**69**	3.06 (1.54)	2.74 (1.32)	-0.32 (0.95)	0.31 (0.01, 0.61)
**Time in MVPA (hours)**	**67**	1.36 (0.64)	1.37 (0.69)	0 (0.5)	**69**	1.36 (0.7)	1.17 (0.58)	-0.19 (0.6)	0.2 (0.03, 0.37)
**Vector magnitude units (counts per minute)**	**67**	266 (170)	266 (185)	-1 (111)	**69**	219 (147)	192 (118)	-28 (98)	39 (4, 75)
**Movement intensity during walking (milligravities)**	**67**	174 (35)	174 (34)	0 (25)	**69**	168 (30)	164 (29)	-4 (21)	5 (-2, 13)
**Total number of activity counts during worn periods (counts*10^3)**	**67**	327 (186)	316 (190)	-10 (123)	**69**	288 (192)	246 (152)	-42 (119)	42 (3, 81)

Data presented as mean (SD) unless otherwise stated.

The estimated mean between-group difference and 95% confidence intervals were calculated by ANCOVA adjusted for baseline value of outcome, left ventricular ejection fraction, age, sex, body mass index, NT-proBNP, New York Heart Association functional class and geographic region.

Abbreviations: LVPA, light-to-vigorous physical activity; MVPA, moderate-to-vigorous physical activity.
